# Improving Enhanced Recovery after Surgery (ERAS): The Effect of a Patient-Centred Mobile Application and an Activity Tracker on Patient Engagement in Colorectal Surgery

**DOI:** 10.1177/15533506241299888

**Published:** 2024-11-08

**Authors:** Sebastiaan L. van der Storm, Marilou Jansen, Malou D. Mulder, Hendrik A. Marsman, Esther C.J. Consten, Frank C. den Boer, Hans D. de Boer, Willem A. Bemelman, Christianne J. Buskens, Marlies P. Schijven, on behalf of the ERAS APPtimize collaborative study group

**Affiliations:** 1 Department of Surgery, 26066 Amsterdam UMC, Location University of Amsterdam, Amsterdam, The Netherlands; 2 571165 Amsterdam Gastroenterology Endocrinology Metabolism, Amsterdam, The Netherlands; 3 1229 Amsterdam Public Health, Digital Health, Amsterdam, The Netherlands; 4 Surgery, 10215 OLVG, Amsterdam, The Netherlands; 5 Surgery, 1170 Meander Medical Center, Amersfoort, The Netherlands; 6 Surgery, University Medical Center Groningen, Groningen, The Netherlands; 7 Surgery, 10177 Zaans Medical Center, Zaandam, The Netherlands; 8 Pain Medicine and Procedural Sedation and Analgesia, 61363 Martini General Hospital Groningen, Partner of the Santeon Healthcare Group, Groningen, The Netherlands

**Keywords:** colorectal surgery, ERAS, recovery, ehealth, mobile health, app

## Abstract

**Background:**

The Enhanced Recovery After Surgery (ERAS) protocol improved perioperative colorectal care. Although the protocol is firmly implemented across hospital settings, there are benefits to gain by actively involving patients in their recovery. The main objective of this study was to investigate whether compliance with selected items in the ERAS protocol could further improve by using a patient-centred mobile application.

**Method:**

This multicentre, randomised controlled trial was conducted between October 2019 and September 2022. Patients aged 18 years or older who underwent elective colorectal surgery, and in possession of a smartphone were included. The intervention group used a mobile application combined with an activity tracker to be guided and supported through the ERAS pathway. The control group received standard care and wore an activity tracker to monitor their daily activities. The primary outcome was overall compliance with selected active elements of the ERAS protocol.

**Results:**

In total, 140 participants were randomised to either the intervention (n = 72) or control group (n = 68). The use of the ERAS App demonstrated a significant improvement in overall compliance by 10%, particularly in early solid food intake by 42% and early mobilization by 27%. Postoperative or patient reported outcomes did not differ between groups.

**Conclusion:**

The smartphone application ‘ERAS App’ is able to improve adherence to the active elements of the ERAS protocol for colorectal surgery. This is an important step towards optimizing perioperative care for colorectal surgery patients and enabling patients to optimize being in control of their own recovery. Trial registration: ERAS APPtimize, NTR7314 (https://trialsearch.who.int/Trial2.aspx?TrialID=NL-OMON29410).

## Introduction

To optimize outcome for patients having to undergo colorectal surgery, the Enhanced Recovery After Surgery (ERAS) Study Group published the first evidence-based consensus protocol for colorectal surgery.^1^ The ERAS protocol outlines the importance of a multidisciplinary and multimodal approach and consists of 24 core elements throughout the colorectal pathways.^2^ All elements work together in an effort to reduce surgical stress, maintain postoperative physiological function, and enhance mobilization after surgery,^3-7^ resulting in a faster recovery, shorter hospital stay, and reduced rates of morbidity.^8-10^

High adherence to the ERAS protocol is significantly associated with markedly improved outcomes, such as shorter hospital stay, lower rates of postoperative complications, reduced 30-day morbidity, and lower readmission rates.^11-14^ However, local implementation of ERAS protocols differ across medical centres. Even when clinical pathways are based on the same ERAS guidelines, implementation of the protocol and outcomes vary.^15^ Protocol adherence were 69%, 72% and 53% during the preoperative, perioperative and postoperative phase respectively.^16^ ERAS protocol compliance may be most essential in the early postoperative phase, as it stimulate early mobilization and resumption of oral intake, avoid discharge delay and minimize the overall risk of complications.^16^

ERAS elements can be categorized as requiring contribution from health care providers (passive elements), patients (active elements), or both (passive/active elements).^17^ The provider-initiated part of the pathway include most ERAS elements which usually has high adherence.^3^ The elements of the ERAS protocol that require patient involvement have the poorest compliance. There are benefits to gain here, as patient empowerment plays an essential part in improving patient adherence.^18,19^

In recent years, mobile health care applications (apps) and wearables have emerged as strategies to improve patients’ adherence to treatment.^20-23^ Apps can provide information, stimulate desired behaviour, enhance self-efficacy and empower patients allowing patients to take an active role in their own health care.^24-26^ Several apps for postoperative recovery have been described in literature, however, the level of evidence and outcomes were varying.^23^ The “ERAS App” is an innovative app which combines stimulation of patient involvement in the ERAS with a personalised activity recovery program. The ERAS App offers an engaging approach to involve patients actively in their own care, providing timed information and recovery goals during the perioperative period.^27^ This randomized controlled trial (RCT) was conducted to assess whether the use of a patient-centred app can significantly increase compliance with the active elements of the ERAS protocol in patients undergoing colorectal surgery.

## Methods

### Study Design

The ERAS APPtimize study is a multicenter RCT that was conducted between October 2019 and September 2022 at one academic hospital and four teaching hospitals in the Netherlands. The ERAS protocol was implemented into the care pathways at varying time points and accompanied by locally different adaptations in the centers. The study was approved by the local medical ethics committee of Amsterdam UMC (registration number NL63874.018.17). The study protocol has been previously published.^27^ The trial was prospectively registered on International Clinical Trial Registry Platforms; registration number NTR7314. The study is reported according CONSORT-EHEALTH checklist and the RECOvER Checklist.^28,29^

### Study Population

Patients were eligible if they underwent elective colorectal surgery for either malignant or benign disease, were aged 18 years or older, and were in possession of a smartphone running at least the operating systems iOS 9 or Android 8.0. Patients were excluded if they met any of the following criteria:

• Palliative surgery or surgery performed after neoadjuvant radiotherapy or chemotherapy
• Karnofsky Performance score ≤40
• Inability to understand thex dutch language
• Visual impairment, unless well corrected with visual aids
• Limitations in using mobile applications due to physical or mental impairments,
• Wheelchair-restricted
• Estimated pre-operatively if post-operative adherence to the ERAS protocol is not feasible
• Resection of multiple organs

### Group Allocation and Blinding

After informed consent, patients were randomly assigned (1:1) using Internet block randomization with block sizes of 2, four, and six to either the intervention or the control group. Randomization was stratified by disease (benign and malignant) and age (<50 years and >50 years). Participants, their involved health care professionals, and outcome assessors of study were not blinded to the treatment allocation. Participants were instructed not to tell other patients in their ward if they are assigned to the intervention or control group to avoid societal bias.

### Procedures

**Figure 1.**
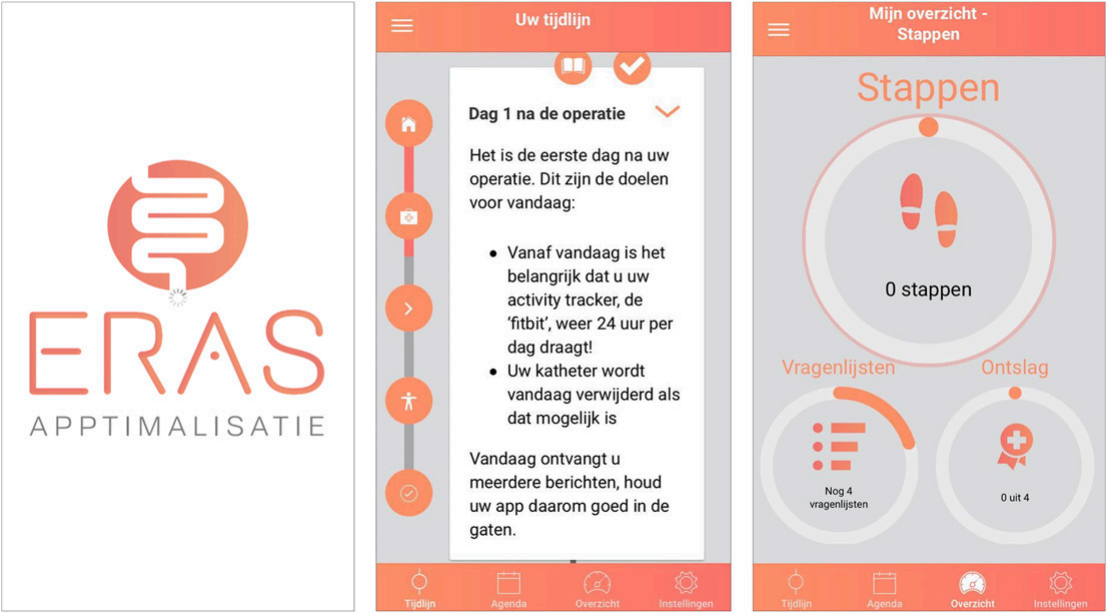
Screenshots of the ERAS App. The app is written in Dutch. First screenshot: Splash screen. Second screenshot: The app generates a timeline based on the operation date which provides information and daily goals to complete. The timeline gives patients an overview of their own care pathway and supports patients to prepare for surgery. If new information or goals are available, push notifications are sent to stimulate patients to adhere to the protocol. Third screenshot: The app’s ‘dashboard’ displays the completion of three subjects: 1) daily activity goal, 2) active ERAS elements, and 3) self-registered questionnaires throughout the entire study.

Participants received care in adherence to the local ERAS protocol in their hospital, which were locally different among study centres. Additionally, the ERAS APPtimize intervention group was supported by the ERAS App spanning from 1-3 weeks preoperatively until 42 days postoperatively. The app was based on the generic ERAS protocol and was designed to educate and actively involve patients in their local perioperative care pathway promoting daily activity. The selected active ERAS elements reported in Table 1 were translated into practical patient-centred features. Push notifications were used to alert patients to new information at specific times to prompt them to complete the necessary actions for each element. All information on the ERAS protocol and required steps could be retrieved and accessed in the app at any time. Daily activity was measured using an activity tracker, starting 7 days prior to surgery or as soon as possible after surgery was scheduled. The average daily step count during the preoperative period of seven days is used to set an individual baseline. During the postoperative phase, daily step goals (Table S1) were offered via push notifications and taken steps were monitored in the app, until 21 days postoperatively. ERAS elements completion checklist and questionnaires are also completed through the app. In study setting, participants had access to the app, as an access code were provided by the research team or health care providers. Participants received instructions at the treatment allocation, had the option the contact the research team for technical support if needed, and used the app according to their own preferences, without any intervention of the research team. Figure 1 displays the app layout. The ERAS App is CE-marked (NL-CA002-2019-47000), complies with the General Data Protection Regulation, and follows ISO 27001 data and security guidelines.^30^

**Table 1.** The Presentation and Scoring of the Selected Active ERAS Elements.

Selected active ERAS elements	Presentation in application	Scoring	
1. Preoperative nutritional screening and, as needed, assessment and nutritional support	SNAQ scoring tool; information	SNAQ ≥3 + no dietician visit	0
		SNAQ ≥3 + dietician visit	1
		SNAQ ≤2	1
2. Preoperative carbohydrate treatment^a^	Push reminders to finish carbohydrate treatment; checklist	No	0
		Yes (1 or 2 bottles)	1
		Tube feeding	1
		Standard use of nutridrink	1
3. Early mobilization	Information; tailored daily goals and reminders; display of progress	No mobilization on day 1	0
		Mobilization on day 1	1
4. Early intake of oral fluids and solids	Information; tailored daily goals and reminders; display of progress	No sufficient oral intake on day 1	0
		Sufficient oral intake on day 1	1
5. Early removal of urinary catheters^a^	Information, checklist	Not removed on day 1	0
		Removed on day 1	1
		No catatherer	1
6. Use of laxatives	Information, checklist	No laxatitives day 1-3	0
		Laxatitives day 1-3	1

^a^The element was not present in all local ERAS protocols of the participating hospitals and therefore was not included in the calculation for overall compliance for these hospitals. Each hospital had a minimum of 5 active elements. Abbrevations: SNAQ Short Nutritional Assessement Questionnaire.

**Figure 2.**
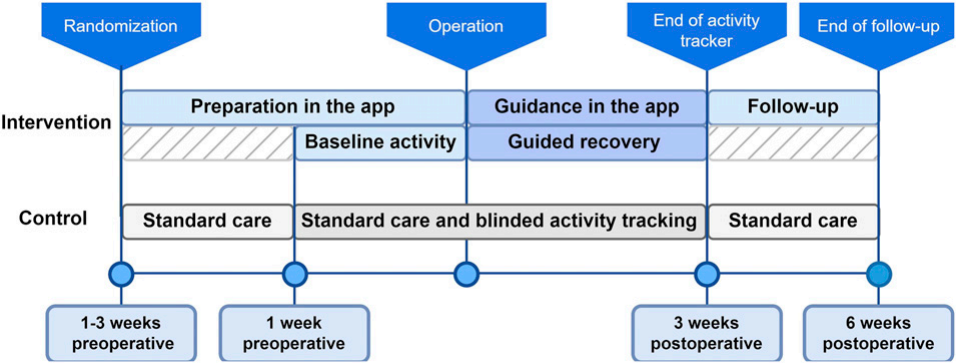
Flowchart of intervention and control group.

Participants assigned to the control group received the usual care following the local ERAS protocol and were given a blinded activity tracker to monitor activity. Participants received a paper booklet containing the ERAS elements completion checklist and questionnaires. They were instructed to complete the checklists once a day and the questionnaires according to the time points shown in Table S2. Figure 2 illustrates the study pathways for both groups.

### Outcome

The primary outcome was overall average compliance with selected active ERAS elements (Table 1). To correct for cofounding on digital (il-)literacy, participants completed a questionnaire on use of apps and their mobile proficiency. Secondary outcome measures were postoperative outcomes, such as length of hospital stay (LOS), complications, readmissions, and reinterventions, as well as patient-reported outcomes (PROMs), including quality of life (measured with WHOQOL-BREF), disability (measured with WHODAS 2.0), and satisfaction with the app (measured using a self-developed questionnaire).^31,32^ Additionally, the activity was assessed from day −7 to surgery, until day 21 post-surgery.

### Statistical Analysis

The sample size was calculated based on a compliance rate to active ERAS elements of 57% in a previous study and the hypothesis that the ERAS App would increase patient compliance to 62%.^17^ Using a 2-sided alpha of 0.05, with 90% power and a standard deviation of 9, 140 patients were estimated to be required for the study. Data were analysed according to protocol analysis.

Statistical analyses were conducted using IBM SPSS version 28.0. Baseline characteristics were summarized using descriptive statistics and compared between the intervention and control groups and between the included and excluded patients. Continuous normally distributed variables were reported as mean ± standard deviation, and non-normally distributed continuous variables were reported as median and interquartile range (IQR). Distributions were evaluated using visual inspection of histograms. Categorical variables were presented as frequencies and percentages. Independent t-tests, Mann-Whitney U tests, Chi-squared tests, and Fisher’s exact tests were used to assess differences between groups as appropriate. A two-tailed *P*-value ≤0.05 was considered statistically significant.

The selected active ERAS elements were dichotomously scored as being fully complete or incomplete. The overall compliance is the average of all individual completion percentages. If a specific ERAS element was not present in the local pathway, it was not included into the calculation of overall compliance for these hospitals. Multivariate linear regression with stepwise backward selection was used to account for potential confounding and stratifying factors.

The extent of surgery was categorized as either being major or minor, with minor surgery defined as stoma creation/removal combined with an enterocutaneous fistula correction and major surgery including all the other operations. Postoperative activity was analysed using a Toeplitz linear mixed model.

PROMs were only included in the analysis if the patient completed >80% of the questionnaire per domain. Missing data were corrected using the participants’ mean of the (domain of the) PROM, if missing values were <20% within that scale.

## Results

**Figure 3.**
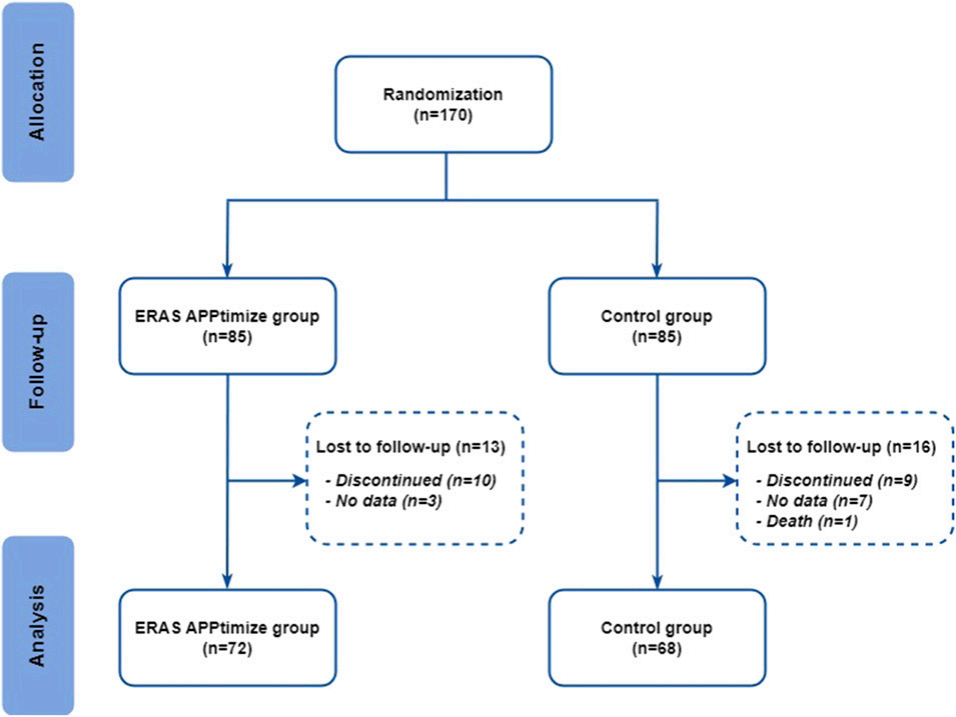
Treatment assignment and study flow.

**Figure 4.**
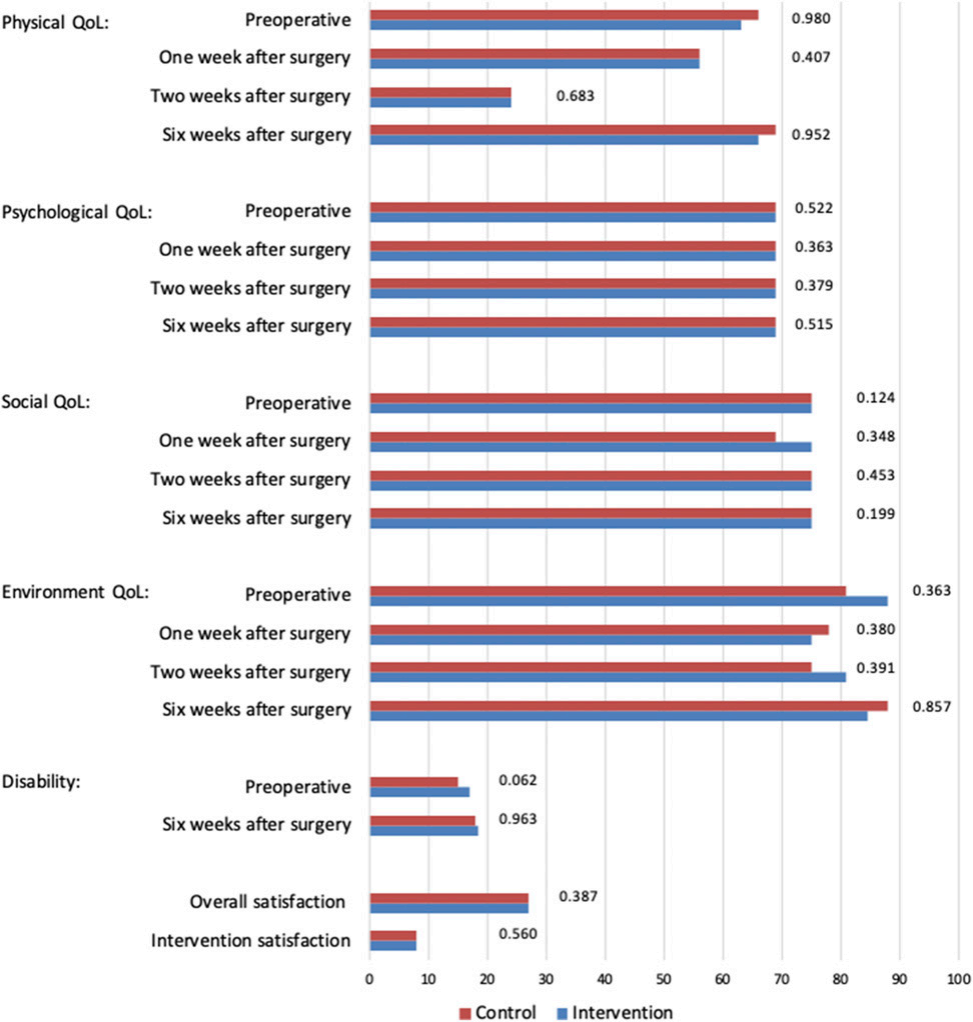
Patient reported outcomes. All reported outcomes are median values. Domains of quality of life are measured in a 0-100 scale, Disabilities is measured in a 12-60 scale, Overall satisfaction is measured in a 7-35 scale, and intervention satisfaction is measured in a 2-10 scale. Abbreviations: QoL = quality of life.

A total of 170 participants provided informed consent and were randomized. Of these participants, respectively 72 and 68 patients were analysed in the intervention and control groups and 30 participants were lost to follow-up (Figure 3). The baseline characteristics of the participants, presented in Table 2, were similar between the 2 groups, with a predominantly male population (n = 77, 54.9%), a median age of 57 years, and a majority of malignant diagnoses (n = 84, 60.1%). Despite randomization, diverticulitis was significantly more prevalent in the control group (*P* = 0.044). Minimally invasive surgery was the predominant mode of surgery in both groups (n = 128, 91.5%), and both groups had sufficient scores on the mobile proficiency questionnaire. Baseline PROM’s are reported in Figure 4.

**Table 2.** Baseline Characteristics of Included Participants.

	Control group (n = 68)	APPtimize group (n = 72)	*P*-value
Male sex	36 (52.9%)	41 (56.9%)	0.634
Age (years)^a^	60 (49 - 68)	55 (44 - 68)	0.249
ASA classification			0.083
1	10 (14.7%)	8 (11.1%)	
2	43 (63.2%)	57 (79.2%)	
3	15 (22.1%)	7 (9.7%)	
Karnofsky performance scale^a^	90 (90 - 100)	100 (90 - 100)	0.630
BMI (kg/m^2^)^a^	25.05 (22.00 – 28.85)	24.00 (22.00 – 27.79)	0.379
Smoking			0.777
<5 pack years n (%)	45 (66.2%)	46 (63.9%)	
≥5 pack years n (%)	23 (33.8%)	26 (36.1%)	
Alcohol (units/week)^a^	1.0 (0.0 – 4.0)	2.0 (0.0 – 6.8)	0.167
Indication			0.562
Benign, n (%)	26 (38.2%)	31 (43.1%)	
Ulcerative colitis	3 (4.4%)	8 (11.1%)	0.277
Diverticulitis*	6 (8.8%)	1 (1.4%)	0.044
Morbus crohn	15 (22.1%)	19 (26.4%)	0.550
Slow transit	1 (1.5%)	1 (1.4%)	0.968
Other	0 (0%)	2 (2.8%)	0.166
Malignant, n (%)	42 (61.8%)	41 (56.9%)	
Colon cancer	20 (29.4%)	16 (22.2%)	0.331
Recto sigmoid cancer	1 (1.5%)	1 (1.4%)	0.968
Rectum cancer	15 (22.1%)	16 (22.2%)	0.981
Sigmoid cancer	5 (7.4%)	6 (8.3%)	0.829
Other	2 (2.9%)	3 (4.2%)	0.696
Procedure			0.617
Minimal-invasive	63 (92.6%)	65 (90.3%)	
Open surgery	5 (7.4%)	7 (9.7%)	
Extent of surgery			0.534
Minor	11 (16.2%)	9 (12.5%)	
Major	57 (83.8%)	63 (87.5%)	
Type of hospital			0.238
Teaching	37 (54.4%)	40 (55.6%)	
Academic	31 (45.6%)	32 (44.4%)	
Mobile proficiency (MDPQ)^a^^b^	38.5 (33.5 – 40.0)	38.5 (32.5 – 40.0)	0.949

^a^Values are median (IQR).

^b^MDPQ Mobile Device Proficiency Questionnaire (scale 8-40).

Abbreviations: ASA American Society of Anaesthesiology; BMI Body Mass Index; IQR inter quartile range.

### Compliance with the ERAS Protocol

Patients in the intervention group had a higher compliance of 10% (76.4%) than patients in the control group (66.4%) (*P* = 0.003) (Table 3). This was mainly due to improved compliance with the early intake of solid foods on day 1 (42%; *P* < 0.001) and start with early mobilization on day 1 (27%; *P* < 0.001). These 2 elements were also higher from day 2 to day 7 after surgery. After adjusting for confounding factors in multivariate linear regression analysis, the improvement in compliance was similar (Table 4). The analysis identified an academic hospital, high Karnofsky score, poor physical health score, and high disability score as being confounders.

**Table 3.** Compliance With the Active ERAS Elements.

	Control group (n = 68)	APPtimize group (n = 72)	*P*-value
Nutritional screening and assessment	89.7 (30.6)	91.7 (27.8)	0.690
Carbohydrate loading	54.4 (50.2)	50.0 (50.4)	0.601
Early mobilization	66.2 (47.7)	93.1 (25.6)	<0.001
Early intake of solid food	45.6 (50.2)	87.5 (33.3)	<0.001
Urinary catheter removed	64.7 (48.1)	63.9 (48.4)	0.920
GI stimulation (laxatives)	77.9 (41.8)	72.2 (45.1)	0.435
Total score	66.4 (23.7)	76.4 (16.7)	0.003

Mean compliance of the group in percentage. Abbreviations: GI Gastro-intestinal.

**Table 4.** Multiple Linear Regression Analysis Compliance to ERAS Protocol.

	B	SE B	95.0% CI	*P*-value
Constant	0.245	0.285	−0.318 – 0.808	0.391
ERAS app	0.095	0.041	0.030 - 0.161	0.005
Age, under 50 years^a^	−0.018	0.043	−0.098 - 0.063	0.666
Benign diagnosis^a^	−0.001	0.033	−0.086 - 0.085	0.989
Teaching hospital	0.163	0.038	0.088 - 0.238	<0.001
Karnofsky performance scale at baseline	0.008	0.003	0.002 - 0.013	0.007
Physical QoL at baseline	−0.003	0.001	−0.005 - 0.000	0.030
Disability at baseline	−0.009	0.004	−0.016 to −0.002	0.013

^a^Stratification factor in randomization. Abbreviations: B Beta coefficient for compliance to ERAS protocol; SE Standard Error; CI Confidence Interval; QoL = quality of life.

### Secondary Outcomes

**Figure 5.**
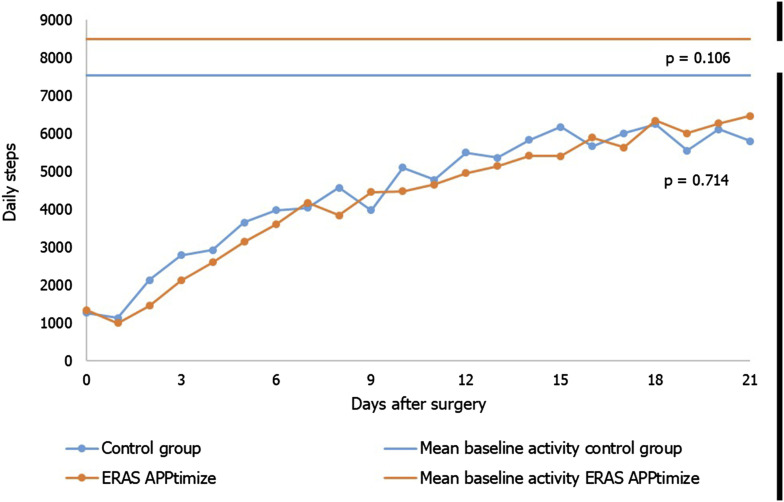
Postoperative activity, measured in steps taken per day.

The median hospital stay was 5 days for patients in both groups. Complications were not reported to be significantly different between the control and intervention group (23.5% 26.4%, *P* = 0.736). The intervention group demonstrated a reduction in reported VAS pain score at day 7 (2.5 vs 3.3 *P* = 0.021). The other postoperative outcomes were comparable between groups (Table 5). The PROMs quality of life, disabilities and satisfaction were similar in both groups (Figure 4). The activities of both groups are presented in Figure 5. Although not statistically significant, preoperative activity did increase by 946 daily steps with the use of the ERAS app (8491 compared to 7545; *P* = 0.106). Postoperative activity in both groups was comparable. However, the intervention group became increasingly active in the last few days of their activity follow-up.

**Table 5.** Postoperative Outcomes.

	Control group (n = 68)	APPtimize group (n = 72)	*P*-Value
Length of hospital stay (days)^a^	5.00 (4.00 – 7.75)	5.00 (4.00 – 6.75)	0.997
Pain (VAS)			
Day 1	4.7 (2.1)	4.9 (2.0)	0.655
Day 2	4.2 (2.1)	4.1 (2.0)	0.771
Day 3	3.6 (2.2)	3.7 (1.9)	0.773
Day 4	3.8 (2.3)	3.5 (1.9)	0.477
Day 5	3.7 (2.5)	3.0 (2.0)	0.113
Day 6	3.6 (2.1)	3.1 (2.0)	0.184
Day 7	3.3 (2.1)	2.5 (1.5)	0.021
Complications	16 (23.5%)	19 (26.4%)	0.736
Ileus	2 (2.9%)	4 (5.6%)	0.445
Gastroparesis	2 (2.9%)	4 (5.6%)	0.445
Anastomotic leakage	4 (5.9%)	9 (12.5%)	0.178
Stoma obstruction	1 (1.5%)	0 (0%)	0.302
Urinary tract infection	2 (2.9%)	2 (2.8%)	0.954
Electrolyte imbalance	2 (2.9%)	2 (2.8%)	0.954
Surgical site infection	1 (1.5%)	3 (4.2%)	0.339
Other, n (%)	6 (8.8%)	4 (5.6%)	0.453
Reintervention	4 (5.9%)	7 (9.7%)	0.523
Readmission	9 (10.6%)	13 (15.7%)	0.228

^a^Values are median (IQR).

## Discussion

The ERAS protocol has improved perioperative care for patients undergoing colorectal surgery. However, challenges persist in optimizing patient engagement and compliance, prompting exploration into innovative mobile health care solutions. This study investigated the effectiveness of a patient-centred app, the ERAS App, designed to enhance patient education, participation and activation within the ERAS colorectal pathway.

The ERAS App demonstrated a significant improvement in overall compliance with selected active ERAS elements by 10% (*P* = 0.003), particularly in early solid food intake by 42% (*P* < 0.001) and early mobilization by 27% (<0.001). Other active ERAS elements remained unchanged, as these elements relies partially on health care providers. Although the postoperative pain was reduced at day 7, the study did not demonstrated improvement in other clinical outcomes. It should be considered that the study was not powered on clinical outcomes as it had already seen significant enhancements since the introduction of the ERAS protocol. Improved adherence to active ERAS elements might translate into clinical benefits in larger study populations. Additionally, the quality of implementation of the ERAS protocol may have varied among health care providers or institutions, leading to inconsistent results across study sites. This highlights the need for standardized implementation and continuous monitoring to ensure protocol effectiveness.

Furthermore, the ERAS App did not improve patient-reported outcomes (PROMs). It is possible that increased adherence to the ERAS protocol may not have a direct impact on PROMs or a potential social desirability bias in self-reported questionnaires could have influenced the observed outcomes.^33^ The ERAS App did not lead to improved postoperative activity. Unusually high baseline activity levels (eg, 23 000 steps per day) were reported in both groups, demonstrating the preoperative motivation which have led to an unrepresentative baseline level. Not all participants had optimal postoperative activity goals, as the baseline measurement may have been too short or goals may not have been sufficiently challenging. The intervention group’s increased activity in the final days suggests that the follow-up period might have been too short to capture sustained improvements.

Several limitations to this study need to be addressed. The exclusion of patients undergoing palliative surgery, surgery after neoadjuvant chemotherapy or radiotherapy, or multiple organ resections, may have resulted in a selection bias. These patients may benefit the most from the app, and their exclusion may underestimate the true impact of the app. In addition, the screening process was not thoroughly registered in all study sites, which inhibited presenting complete screening data in Figure 3 as this may lead inaccurate conclusions about the selection bias. Study results should be interpreted with this context in mind. Furthermore, non completing participants had significantly more complications (Table S4), which suggests that the ERAS App may not be optimal for patients with complications. This highlights the need for further research. It is important to note that patients in the control group may have been more actively participating in the ERAS care pathway compared to their peers as. This may have resulted in a decreased compliance difference between the 2 study groups.

Despite the demonstrated effect of the ERAS App, opportunities for further optimization were identified. Dynamic features catering to individual recovery progress and adapting to postoperative complications hold promise. However, it’s important to exercise caution when integrating individual recovery progress because the more personalized the intervention, the less evidence there is to support its overall effectiveness. The integration of prehabilitation with the ERAS App emerges as a potential strategy to improve clinical outcomes.^34^ Future research should delve into the feasibility and efficacy of incorporating dynamic features or prehabilitation within the ERAS pathway through the ERAS App. Additionally, exploring barriers and facilitators to the app’s implementation in clinical practice can inform strategies for enhancing its adoption and utilization. Overall, further research and development of the ERAS App can lead to better patient engagement, adherence to the ERAS protocol, and improved clinical outcomes.

## Conclusion

The ERAS App successfully increases patient compliance to the ERAS protocol by actively involving patients into their own ERAS care. Although the ERAS App was unable to demonstrate improved patient-related and clinical outcomes, the app is an important step towards optimizing perioperative care for colorectal surgery patients and enabling patients to optimize being in control of their own recovery. Further research and development are necessary to identify ways to improve the app’s efficacy and impact on patient outcomes.

## Supplemental Material

Supplemental Material - Improving Enhanced Recovery after Surgery (ERAS): The Effect of a Patient-Centred Mobile Application and an Activity Tracker on Patient Engagement in Colorectal Surgery Supplemental Material for Improving Enhanced Recovery after Surgery (ERAS): The Effect of a Patient-Centred Mobile Application and an Activity Tracker on Patient Engagement in Colorectal Surgery by Sebastiaan L. van der Storm, Marilou Jansen, Malou D. Mulder, Hendrik A. Marsman, Esther C.J. Consten, Frank C. den Boer, Hans D. de Boer, Willem A. Bemelman, Christianne J. Buskens, Marlies P. Schijven, on behalf of the ERAS APPtimize collaborative study group in Surgical Innovation
