# Supplementary material for: Improving Enhanced Recovery after Surgery (ERAS): The Effect of a Patient-Centred Mobile Application and an Activity Tracker on Patient Engagement in Colorectal Surgery
Source: Surg Innov. 2024 Nov 8;32(1):5–15. doi: 10.1177/15533506241299888 (PMC11736976; doi:10.1177/15533506241299888)
Supplement: Supplemental Material - Improving Enhanced Recovery after Surgery (ERAS): The Effect of a Patient-Centred Mobile Application and an Activity Tracker on Patient Engagement in Colorectal Surgery [file sj-pdf-1-sri-10.1177_15533506241299888.pdf]

Table S1: Timing of the measurements

	T0	T1	T2	T3	T4	T5
Baseline characteristics	X					
Protocol compliance		X	X			
Activity		X	X	X	X	
Postoperative outcomes						X
MDPQ	X					
WHOQoL-bref	X		X	X		X
WHODAS 2.0	X					X
Satisfaction						X

T0: Inclusion, T1: 1 week preoperative, T2: 1 week postoperative, T3: 2 weeks postoperative, T4: 3 weeks postoperative, T5: 6 weeks postoperative.

Abbreviation: WHO-bref World Health Organization Quality of Life Abbreviated Version ; WHODAS 2.0 World Health Organization Disability Assessment Schedule 2.0.
The WHOQOL-BREF questionnaire measures four domains: physical, psychological, social, and environmental, with each category graded on a 5-point scale ranging from 'Very dissatisfied' [1] to 'very satisfied' [5] (21). The total score is calculated according to the questionnaire's protocol and ranges from 0-100 points per domain, with higher scores indicating a higher quality of life. Health-related disability was assessed using the WHODAS 2.0 questionnaire, which grades disability on a 5-point scale ranging from 'no limitations' [1] to 'many limitations' [5] (22). The total score ranges from 12-60 points, with lower scores indicating less limitation due to illness. The self-developed patient satisfaction questionnaire contains questions about satisfaction with perioperative care and the received intervention.

Table S2: Daily step goal

	Days	Daily step goal
Preadmission	-7 untill -1	Registatrion for postoperative step goal
Postoperative	0	50
	1	2.0% * preadmission step mean
	2	6.7% * preadmission step mean
	3 until 5	19.6% * preadmission step mean
	6 until 8	28.9% * preadmission step mean
	9 until 11	38.3% * preadmission step mean
	12 until 14	47.7% * preadmission step mean
	15 until 17	57.0% * preadmission step mean
	18 until 20	66.5% * preadmission step mean
	21	76.0% * preadmission step mean

Table S3: Sub-analysis according to hospital type

		Academic hospital (n=71)	Teaching hospital (n=69)	P - value
Age (years) [a]		52.00 (38.00 – 62.00)	63.00 (55.00 – 71.00)	* <0.001
BMI (kg/m2) [a]		23.00 (21.00 – 27.00)	25.60 (23.30 – 28.80)	* 0.013
Primary diagnosis	Benign, n (%)	47 (66.2%)	10 (14.5%)	
	Malignant, n (%)	24 (33.8%)	59 (85.5%)	
Extent of surgery	Minor, n (%)	16 (22.5%)	4 (5.8%)	* 0.005
	Major, n (%)	55 (77.5%)	65 (94.2%)	
LOS, (days) [a]		6.00 (5.00-10.00)	4.00 (3.00-5.00)	* <0.001
Reintervention		10 (14.1%)	2 (2.9%)	* 0.018
Quality of life	Physical [a]	56.00 (44.00 – 75.00)	69.00 (56.00 – 81.00)	* <0.001
	Psychological [a]	69.00 (56.00 – 81.00)	75.00 (69.00 – 81.00)	
	Social [a]	75.00 (50.00 – 81.00)	75.00 (69.00 – 94.00)	* 0.041
	Environment [a]	81.00 (69.00 – 88.00)	88.00 (75.00 – 94.00)	* 0.012
Disabilities [a]		17.00 (15.00 – 26.00)	15.00 (13.18- 19.00)	* 0.016

Abbreviations: BMI; Body mass index, LOS; length of stay,

**Significant difference between groups*

[a] *Values are median (IQR)*
